# Motor Imagery Impairment in Postacute Stroke Patients

**DOI:** 10.1155/2017/4653256

**Published:** 2017-03-28

**Authors:** Niclas Braun, Cornelia Kranczioch, Joachim Liepert, Christian Dettmers, Catharina Zich, Imke Büsching, Stefan Debener

**Affiliations:** ^1^Neuropsychology Lab, Department of Psychology, University of Oldenburg, Oldenburg, Germany; ^2^Kliniken Schmieder Allensbach, Allensbach, Germany; ^3^Kliniken Schmieder Konstanz, Konstanz, Germany; ^4^Research Center Neurosensory Science, University of Oldenburg, Oldenburg, Germany; ^5^Cluster of Excellence Hearing4All, University of Oldenburg, Oldenburg, Germany

## Abstract

Not much is known about how well stroke patients are able to perform motor imagery (MI) and which MI abilities are preserved after stroke. We therefore applied three different MI tasks (one mental chronometry task, one mental rotation task, and one EEG-based neurofeedback task) to a sample of postacute stroke patients (*n* = 20) and age-matched healthy controls (*n* = 20) for addressing the following questions: First, which of the MI tasks indicate impairment in stroke patients and are impairments restricted to the paretic side? Second, is there a relationship between MI impairment and sensory loss or paresis severity? And third, do the results of the different MI tasks converge? Significant differences between the stroke and control groups were found in all three MI tasks. However, only the mental chronometry task and EEG analysis revealed paresis side-specific effects. Moreover, sensitivity loss contributed to a performance drop in the mental rotation task. The findings indicate that although MI abilities may be impaired after stroke, most patients retain their ability for MI EEG-based neurofeedback. Interestingly, performance in the different MI measures did not strongly correlate, neither in stroke patients nor in healthy controls. We conclude that one MI measure is not sufficient to fully assess an individual's MI abilities.

## 1. Introduction

Stroke is a leading cause of chronic motor impairment in adults. To aid motor recovery, various interventions have been developed [1]. A widely known example is constraint-induced movement therapy or CIMT [2], for which a number of studies have demonstrated improvements in motor and functional outcomes [3]. A severe limitation of CIMT however is that it requires residual movement [4]. Motor imagery (MI) training has been suggested as a promising alternative or add-on therapy to CIMT and other physical therapies (for a review, see [5]). Based on neurofunctional evidence for similar activation patterns during motor execution and MI [6], this intervention seeks for a “backdoor to the motor system” [5]. MI-based activation of sensorimotor areas is thought to support cortical reorganization and thereby to aid motor recovery [7, 8].

Different MI training protocols have been suggested for motor rehabilitation [5, 9]. Common to all of them is the (implicit) assumption that stroke patients can still perform MI or that they are at least able to regain this ability during training. Not much is, however, known about whether stroke patients are able to conduct MI and, if so, whether clinical subgroups differ in their ability to conduct MI. While some studies found MI in general to be impaired after stroke [10], others found specific MI aspects to be impaired [11, 12] or no MI impairment at all [13, 14]. This heterogeneity of results may be partly explained by the different stroke populations investigated. For instance, Liepert et al. [12, 15] found that an impairment of the chronometric aspects of MI is specifically observed in stroke patients with a severe somatosensory deficit. Another important factor potentially contributing to this heterogeneity, however, is the different MI measures used. Whereas some groups used subjective questionnaires [16, 17], others applied objective MI tasks [12–15, 18]. The two most commonly used objective, implicit assessments of MI, are mental chronometry and mental rotation tasks. In mental chronometry tasks, the degree to which imagined and executed movements share similar temporal profiles is quantified [12, 15, 19]. In mental rotation tasks, the participant's ability to identify the laterality of spatially rotated limb pictures is assessed [11]. Another objective way of MI assessment is to investigate the individual's neuronal profile during an explicit MI task [20]. Similar to motor execution, explicit MI results in a decrease in 8–30 Hz oscillatory brain activity over contralateral sensorimotor scalp sites (for a review, see [21]). This pattern, described as event-related desynchronization (ERD), is a reliable neuronal indicator of whether MI is conducted properly or not, and it can be utilized for MI-based neurofeedback training regimes (for a review, see [22]).

The aim of the present study was to better understand how the different MI tasks relate to each other and whether they can be used interchangeably to assess a patient's MI ability. We conducted three different MI tasks in a sample of postacute stroke patients and age-matched healthy controls. By comparing different objective behavioral and electrophysiological MI measures, we addressed three research questions: First, we asked which of the MI tasks indicate MI impairment in stroke patients and whether these impairments are specific to the paretic side. Second, we determined whether MI impairments are related to sensitivity loss and/or severity of paresis. And third, we examined whether performance in the different MI tasks converges in healthy individuals and stroke patients.

## 2. Materials and Methods

### 2.1. Participants

Twenty-three stroke patients and the same number of age-matched healthy controls were recruited for the study (see Table 1 for demographic and clinical data). All participants were required to have normal or corrected-to-normal vision and no known history of a psychiatric disorder. Stroke patients were in a subacute or chronic stroke state (at least 1 month after stroke). Inclusion criterion was a moderate to severe hand paresis due to stroke. Patients were required to have no epileptic seizures, no dementia, and no severe aphasia or neglect that would impair their ability to follow task instructions. Controls were matched for age and sex. None of the participants had previous experience with neurofeedback or MI training. Controls were paid for their participation. All participants gave written informed consent and were naive to the purpose of the study. The experiment was conducted in accordance with the Declaration of Helsinki and approved by the University of Oldenburg ethics committee. Three stroke patients had to be excluded from the statistical analysis, two for failing to follow task instructions and one for withdrawing during the experiment. The corresponding matched control subjects were also excluded.

### 2.2. General Procedure

The MI tasks were conducted in a silent room. Participants sat in front of a table, on which a computer screen was placed. At the beginning of the experiment, the experimenter prepared the EEG measurement, while the participants filled in questionnaires. For stroke patients, a sensorimotor assessment followed. Afterwards, participants first performed the mental chronometry task (5–10 minutes), followed by the mental rotation task (10 minutes), and finally the MI-based neurofeedback task (45 minutes). The different MI tasks were always conducted in this order. The complete session lasted around 2 hours.

### 2.3. Motor Assessment

Upper-limb motor dexterity was assessed with the Box and Block Test (BBT; cf. [23]). The BBT consists of a flat box with two separate compartments, a barrier in the middle, and a number of blocks. Measured is the time needed to pick up the blocks from one compartment, carrying them over the barrier and then dropping them into the other compartment. In the present implementation, the BBT box was 30 cm in width, 38 cm in length, and 4 cm in height, and the barrier protruded 10 cm out of the box. The BBT was oriented on the table in front of the participant such that the barrier was in line with the participant's midsagittal plane. Fifteen wooden blocks (2 cm in diameter) were positioned in a 3 × 5 matrix within the left or right compartment, depending on which side the test was conducted. Upon the experimenter's start signal, the participant started to pick up the blocks one after another and to place each block in the other compartment. For picking up the blocks, the participants had to follow a fixed sequence, beginning with the first block in the most upper row and ending with the last block in the most lower row. For putting the blocks down, no sequence was predefined. Patients performed the task two times on each side, always starting with the paretic side. Mean performance times were separately calculated for paretic and nonparetic sides, and a motor dexterity index was derived by the ratio between both performances. Five stroke patients were unable to perform the BBT with their paretic hand.

### 2.4. Sensitivity Assessment

Sensitivity assessment was separately performed for the paretic and nonparetic body sides and focused on stereognosis, proprioception, and thermoception. Stereognosis was tested by pseudorandomly touching each of the patient's fingers twice. The proportion of correctly classified touches out of the ten touches on each hand was then used as a stereognosis score. Likewise, proprioception was assessed by pseudorandomly moving each of the patient's fingertips either up or down and then calculating the proportion of correctly classified finger movements out of all ten finger movements. Thermoception was tested by interchangeably giving the patient a glass with hot or cold water into the hand (again, 10 times on each side) and then calculating the proportion of correct cold/warm responses out of all responses. An overall sensitivity score for each side was calculated by taking the average of all three individual scores.

### 2.5. Nine-Hole Peg Test

**Figure 1:**
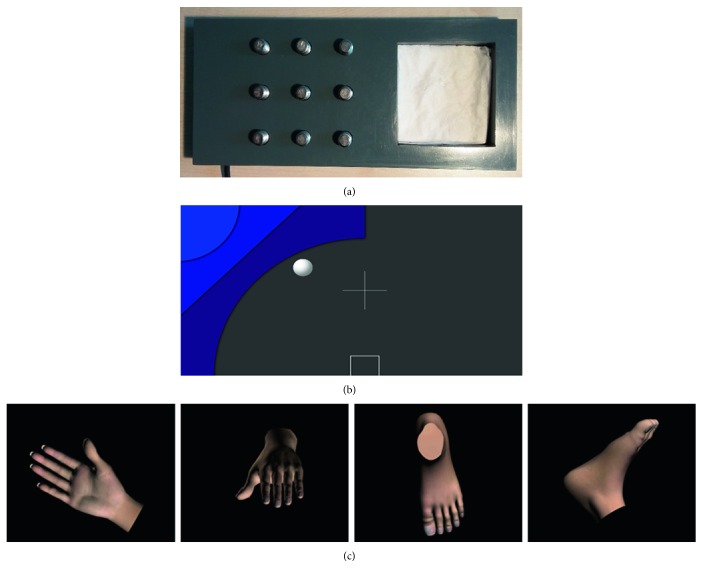
Test material of the different MI tasks. (a) Nine-Hole Peg Test. Participants were required to physically/mentally remove each of the 9 pegs from the holes and put them into the depression. (b) Visualization of the two-dimensional neurofeedback display. The location of the blue shape signaled the hand to be used for MI, and the ball represented the neurofeedback signal. The horizontal ball position is determined by the classification of MI contralateral versus ipsilateral, and the vertical ball position is determined by the classification of contralateral baseline versus contralateral MI. (c) Example stimuli from the limb lateralization task. Left-hand, right-hand, left-foot, or right-foot pictures were presented from varying angles, and participants had to judge their laterality.

To assess the chronometric aspects of MI, the Nine-Hole Peg Test (NHPT) [24] was used. In this task, the participant has to remove nine pegs as quickly as possible out of a pegboard and put them into a container. Depending on the condition, this action is either physically executed or imagined [25]. The performance times of both conditions are then put into a ratio to calculate a mental chronometry score (MC score; see next). In the present study, the NHPT consisted of a plastic console (23 cm in length, 10 cm in width, and 2 cm in height) with a depression on one end of the console and nine holes (arranged in a 3 × 3 matrix) holding the pegs (8 mm in diameter, 3 cm in length) on the other end (Figure 1(a)). Participants performed four runs per hand, two with the executed movement and two with the imagined movement. Test instructions were given along with a brief demonstration. For the execution runs, all participants were instructed to start removing each of the pegs one by one with the “Go” signal given by the experimenter and to put them into the depression. Participants were instructed to remove the pegs as quickly as possible and in a fixed sequence, starting with the first upper-left peg and ending with the last lower-right peg. The end of each run was indicated by the participant saying “Stop.” For the MI runs, the participant was instructed to conduct exactly the same task, but this time, the movements were only mentally performed. Patients were reminded to take the impairment of the paretic hand into consideration when imagining the movement. Before the beginning of the task, all participants were given the opportunity to hold a peg to acquaint themselves with its surface and weight. Each run was timed with a stopwatch from the moment the experimenter said “Go” until the moment the participant said “Stop.” For patients, the NHPT always began with the MI run on the paretic side, and for the matched healthy control, it started with the side corresponding to the paretic side in the matched patient. The pegboard was placed in front of the participant such that the depression was in line with the participant's midsagittal plane and the peg holes were on the paretic body side or the side corresponding to the paretic body side. After the MI run, the physical execution run followed. Then, the pegboard was rotated by 180 degrees such that the peg holes were now on the opposite side and the depression was again aligned to the midsagittal plane. As before, the MI run was performed first, followed by the physical execution run. The procedure was repeated twice, resulting in a total of eight runs. Five stroke patients were unable to perform the physical execution runs with their paretic side. An MC score was calculated for each limb side in accordance with the formula motor execution − motor imagery/motor execution [12, 15]. Values of zero indicate a perfect isochronism between MI and motor execution, whereas values higher than zero indicate that the task was performed faster during MI than during motor execution and values lower than zero indicate that the task was performed slower during MI than during motor execution.

### 2.6. Limb Lateralization Task

For assessing the mental rotational aspects of MI, the limb lateralization task (LLT) was used. In this task, limb pictures are presented from different angles and participants have to judge their laterality [26, 27]. Based on a realistic 3D hand model and a realistic 3D foot model (TurboSquid, Louisiana, USA), limb pictures were created for the left hand, right hand, left foot, and right foot. For deriving the stimulus material, we followed the procedure described by Ter Horst et al. [26], according to which limb pictures are rotated over three axes (in-plane, longitudinal, and in-depth). Examples of the stimuli are shown in Figure 1(c). In total, 136 stimuli were created, with an equal number of stimuli (*n* = 34) for each of the four limbs. Stimulus presentation was controlled with NBS presentation 18.1 (Neurobehavioral Systems Inc., Albany, USA). The 136 pictures were presented in a randomized order. Each trial began with the 1 s fixation cross, followed by the presentation of a picture. As soon as the picture appeared on the screen, participants were asked to indicate as quickly and as accurately as possible whether a left or right limb was shown. Participants responded verbally by saying “left” or “right,” and the responses were directly entered in the ongoing LLT by the experimenter. A verbal response mode was chosen because manual response modes have been shown to interfere with the LLT [28]. As soon as the experimenter had entered a participant's response or when seven seconds had passed, the limb picture disappeared and the screen became blank again until a button pressed by the experimenter started the next trial. No feedback about performance was provided. Participants were not allowed to move their hands throughout the task. For LLT performance evaluation, overall classification accuracies and classification accuracies for each limb side (i.e., true positive rates) were calculated.

### 2.7. MI EEG-Based Neurofeedback

To investigate the neural aspects of MI, a MI EEG-based neurofeedback training was conducted. A modified version of the Graz MI protocol was used [29]. In the used implementation, run in OpenViBE Designer 0.16.2 [30], participants imagine left- or right-hand movements while receiving online neurofeedback about their current ERD pattern. For the present study, an initial calibration block was combined with two subsequent feedback blocks, each block lasting 8 minutes. A block included 20 left-hand MI and 20 right-hand MI trials, presented in a pseudorandomized order. The training block was introduced to acquaint the participants with the overall MI task and calibrate the classifiers for the first neurofeedback block. Each trial started with a fixation cross, which was joined after 2.5 s by a triangle-like geometric shape (Figure 1(b)), appearing either on the upper-left or upper-right side of the screen for 5 s. The spatial location of the shape indicated the hand to be used for the MI task. Participants were instructed to kinesthetically imagine two flexion-extension movements with the respective hand from a first-person perspective while the shape was on-screen. After the MI period, a blank screen was presented for 4.5 to 6 s (in steps of 0.5 s), indicating the participant to relax. In the two neurofeedback blocks, the display included a ball that was moving on the screen during the 5 s MI periods. The position of the ball reflected the neurofeedback signal and was determined by two classifier outputs. Whereas the vertical position of the ball was determined by classification of the contralateral ERD during MI versus baseline, the horizontal position resulted from the classification of contra- versus ipsilateral ERD during MI (i.e., ERD laterality). To avoid accidental movements, the experimenter visually inspected the participant's hands throughout the neurofeedback task and, whenever necessary, reminded the participant to not actually move. Two stroke patients were unable to follow the neurofeedback task due to cognitive impairment, and two additional datasets (one stroke and one control dataset) had to be excluded due to poor signal quality.

#### 2.7.1. Classifier Training and Online Data Flow

EEG data were collected with a wireless EEG system (mBrainTrain GmbH, Belgrad, Serbia) from 24 scalp sites using an elastic cap (EASYCAP, Herrsching, Germany). The electrode montage was a subset of the 10–20 systems and included positions FP1, FP2, F7, F8, FZ, FC1, FC2, T7, C3, CZ, C4, T8, TP9, CP5, CP1, CPz, CP2, CP6, TP10, P3, PZ, P4, O1, and O2. FCz served as reference (CMS) and AFz as ground (DRL). The continuous EEG signal was recorded with OpenViBE acquisition server 1.1.0 [30] with a sampling rate of 500 Hz. To provide neurofeedback, EEG data were analyzed on site using a two-step procedure. The first step was performed between blocks using OpenViBE and EEGLAB [31]. To derive individual spatial filter coefficients, the EEG data were offline band-pass filtered (8–30 Hz) and segmented from 0.5 to 4.5 s, relative to the onsets of the MI periods. After artifact rejection (pop_jointprob.m, SD = 3), the segments were submitted to common spatial pattern (CSP) analysis. Given two time windows of a multivariate signal, this algorithm finds spatial filters that maximize the variance for one class and simultaneously minimize the variance for the other class (for reviews, see [32, 33]). To derive one CSP filter for left-hand MI and one for right-hand MI, the first four and last four CSP filters (promising high class discriminability) were evaluated for their spatial topography and associated time course. The two CSP filters best reflecting the expected sensorimotor cortex activity for left- and right-hand MI were selected. The CSP coefficients were exported to OpenViBE. Here, the EEG data were temporally filtered (8–30 Hz), spatially filtered using the two chosen CSP filters and segmented into baseline (7–3 s before graphic onset) and MI intervals (0.5–4.5 s after graphic onset) for the right and left hand separately. These segments were then subdivided into 56 time bins, each containing a 1 s time window, shifted in time by 62.5 ms. From each bin, one training example was calculated by taking its log variance across time, resulting into two feature values. Different subsets of these training examples were then carried over to linear discriminant analysis (LDA) to derive three classifiers (left MI versus right MI, right MI versus baseline, and left MI versus baseline). For the left MI versus right MI classifier, the examples resulting from the MI periods were used; for the right MI versus baseline classifier, the examples resulting from the right MI periods and its preceding baseline periods; and for the left MI versus baseline classifier, the examples resulting from the left MI periods and its preceding baseline periods. For the second step of the on-site analysis of the online data flow, feature values were derived in the same manner as during classifier training, whereby the classifier was consulted every 62.5 ms, always operating on the most recent 1-second EEG segment. Borders of the feedback display were kept constant within each block and were defined as the upper quartile of the classifier outputs from the previous block.

#### 2.7.2. Neurofeedback Performance Evaluation

Neurofeedback performance evaluation was carried out offline on artifact-corrected EEG data. EEG artifact attenuation was performed using extended infomax independent component analysis (ICA) [31, 34] on data aggregated across all three blocks. Artifactual independent components were identified by visual inspection, and the corresponding activity was removed by excluding the respective components from back projection. The ICA-corrected data were segmented from 0.5 to 4.5 s, relative to the onsets of the MI and baseline periods. Segments containing unique, nonstereotyped artifacts (e.g., swallowing and movements of electrode cable) were identified by built-in EEGLAB functions (pop_jointprob.m, SD = 3; pop_rejkurt.m, SD = 3) and rejected. The remaining segments were 8–30 Hz band-pass filtered and then sorted into training and feedback segments. Based on the segments of the training block only, two classifier models were calculated, one for baseline versus left MI classification and one for baseline versus right MI classification. To derive the baseline versus left MI classifier, the baseline and left MI segments were first submitted to the CSP algorithm and the most physiologically plausible CSP component was selected following the same procedure as described before. Then, the resulting CSP-filtered 4 s segments were further segmented into four consecutive 1 s time intervals, and for each interval, a feature value was calculated by taking its log variance. For each segment (baseline or left MI period), the number of feature values was thus four, and a regularized LDA (as implemented in Lotte and Guan [35]) was trained on the resulting feature vector. To obtain the baseline versus left MI classification accuracy during feedback, this classifier was then applied to the respective segments of the two (collapsed) feedback blocks. Exactly the same procedure was conducted for the baseline versus right MI classifier, with the exception that, here, the baseline and right MI periods were used.

#### 2.7.3. ERD Analysis

ERD analysis focused on temporospectral differences between the different experimental conditions and hemispheres. EEG data artifact attenuation and removal were done as described for the neurofeedback performance evaluation, but this time, the EEG data was segmented from −2.5 to 7.5 s, relative to the onset of the MI periods. A time-frequency (TF) analysis was performed on these segments using a continuous Morlet wavelet transform [36, 37]. The obtained frequency bins ranged from 5 to 35 Hz in 1 Hz frequency steps. To account for edge artifacts, TF data were only considered from −2.2 s to 7.2 s, relative to MI onset. Percent power change relative to baseline power was calculated. For each frequency bin, this was achieved by squaring its belonging data, scaling it to decibels (10 × log10), and calculating its change in power, relative to the first 1.5 s mean baseline power. For the statistical analysis, ERD values were extracted for electrode sites C3 and C4 by taking the mean percent log power changes across trials between 8 and 30 Hz, averaged over a 3.5 s time interval beginning 500 ms after MI onset. These electrode sites were used because it has been shown that for the group average, these locations show the strongest ERD effects [38]. ERD lateralization was calculated as the difference between the contralateral (C3 or C4) and ipsilateral (C4 or C3) ERD with respect to the hand used.

### 2.8. Statistical Analysis

The main statistical analyses focused on four dependent variables: MC score, LLT performance, classification accuracy, and ERD lateralization. To statistically test whether LLT performance and classification accuracies were above chance level, a binomial statistic with a confidence limit of *p* = 0.05 was used [39].

To address the first research question, a 2 × 2 analysis of variance (ANOVA) with the between-subject factor Group (stroke versus control) and the within-subject factor Side (paretic versus nonparetic) was conducted. All measurements were assigned to the paretic or nonparetic side—regardless of whether the paresis was on the left or right hand. For the matched controls, the corresponding assignment was used, meaning that here the term “paretic” was assigned to the side for which the paresis was evident in the respective stroke patient. For example, for a control matched to a patient with a paresis of the left hand, a left-hand movement was assigned to the “paretic” side and a right-hand movement to the “nonparetic” side. The Group × Side mixed-model ANOVA was performed for each dependent variable. Significant interactions were followed up by post hoc *t*-tests.

The second research question focused on the stroke data of the paretic side. To test for an influence of sensibility loss, stroke patients were grouped into those with sensibility loss and those without sensibility loss. This grouping was based on the sensibility score with only patients achieving the sensibility score's maximum (10) being assigned to the group without sensibility loss. Likewise, to test for the influence of severity of motor loss, patients were divided into those with severe motor impairment and those with less severe motor impairment, as based on a median split of the motor dexterity score. A summary of the demographics and clinical characteristics of the four different subgroups is given in Table 2. For each of the four main experimental variables, two *t*-tests were calculated, one comparing patients with sensitivity affected versus sensitivity unaffected and one comparing patients with severe paresis versus moderate paresis. These *t*-tests will be referred to as sensitivity comparisons and paresis comparison, respectively.

In order to identify the possible association between the MI measures, Pearson's *r* correlation coefficients were calculated for each pair of measures. Correlation coefficients were separately calculated for each group and limb side (paretic versus nonparetic).

## 3. Results

### 3.1. Group Differences in MI Performance and Paresis Side Specificity

Our first study aim was to investigate which measure derived from three objective MI tasks indicates MI impairment in stroke patients and whether these impairments are specific to the paretic side.

**Figure 2:**
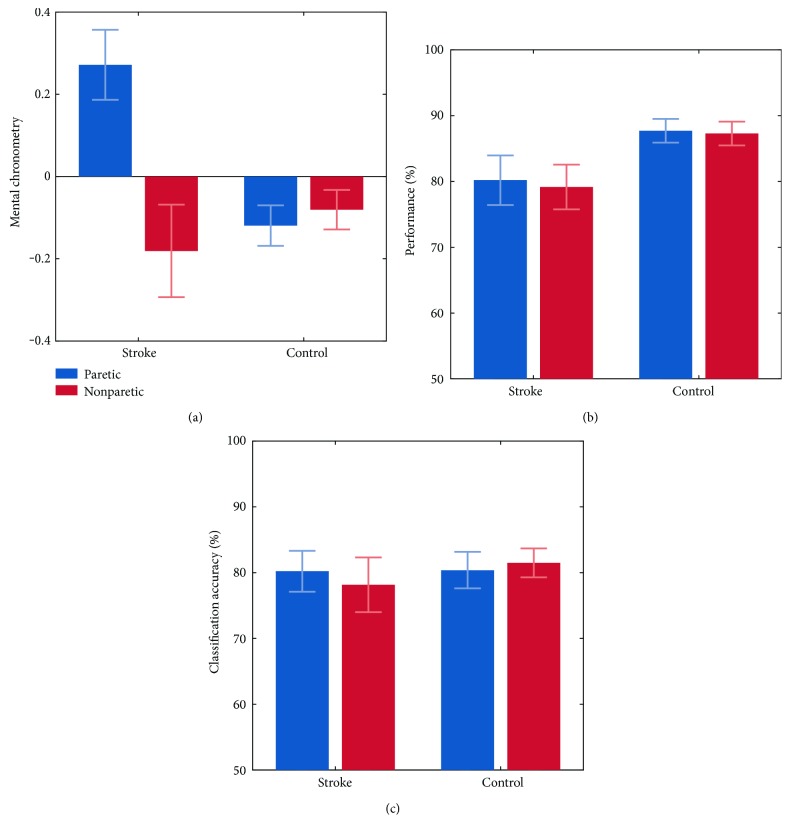
Group differences in MI performance and paresis side specificity. Error bars represent one standard error.

Starting with the NHPT results (Figure 2(a)), a positive MC score, that is, a shorter time to complete the task during MI than during motor execution, was observed for the stroke-paretic condition (*M* = 0.27; SD = 0.32), whereas negative MC scores were observed for the stroke-nonparetic (*M* = −0.18; SD = 0.46), control-paretic (*M* = −0.11; SD = 0.21), and control-nonparetic conditions (*M* = −0.08 SD = 0.21). The 2 × 2 mixed-model ANOVA revealed a significant main effect of Group (*F*(1,33) = 5.54; *p* < .024), a significant main effect of Side (*F*(1,33) = 12.59; *p* < .001), and an interaction between Group and Side (*F*(1,33) = 18.88; *p* < .001). Post hoc *t*-tests were significant for stroke-paretic versus stroke-nonparetic (*t*(30) = 3.16; *p* = .003) and stroke-paretic versus control-paretic (*t*(33) = 4.23; *p* < .001), but not for control-paretic versus control-nonparetic (*t*(38) = −0.56; *p* = .573) and stroke-nonparetic versus control-nonparetic (*t*(35) = −0.87; *p* = .387).

LLT performance (Figure 2(b)) was significantly above chance level (*α* = 0.05) in 76 of the (20  patients + 20  controls) ∗ 2  hands = 80  cases. LLT performance amounted to an average of 80.20% in the stroke-paretic condition (SD = 15.91), 79.16% in the stroke-nonparetic condition (SD = 14.43), 87.70% in the control-paretic condition (SD = 8.08), and 87.29% in the control-nonparetic condition (SD = 8.10). A 2 × 2 mixed-model ANOVA revealed a significant main effect of Group (*F*(1,36) = 4.41; *p* < .042) in that the stroke group performed lower (*M* = 79.68%; SD = 14.98) than the control group (*M* = 87.50%; SD = 7.99). No main effect of Side (*F*(1,36) = 0.39; *p* = .532) and no Group × Side interaction (*F*(1,36) = 0.07; *p* = .788) were found.

Overall offline classification accuracies for the neurofeedback task are depicted in Figure 2(c). Classification accuracies were significantly above chance level (*α* = 0.05) in 68 of the (17  patients + 19  controls) ∗ 2  hands = 72  cases. In all of the four conditions (stroke-paretic, stroke-nonparetic, control-paretic, and control-nonparetic), the overall classification accuracies across subjects were around 80%. The ANOVA did not reveal any significant main effect of Group (*F*(1,35) = .189; *p* < .666) or Side (*F*(1,35) = .081; *p* = .777). Also, there was no significant Group × Side interaction (*F*(1,35) = 0.93; *p* = .340).

**Figure 3:**
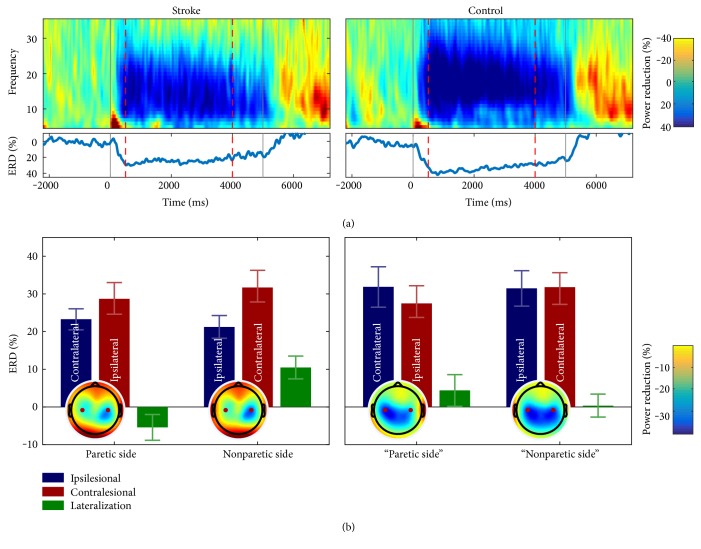
Event-related desynchronization during MI-based neurofeedback. (a) Time frequency plots of the contralateral electrode site (C3 or C4) showing percentage change in power from baseline for MI with the paretic side. MI started at time point zero and was performed for 5 seconds (solid vertical lines). The two dashed vertical lines indicate the time interval used for the statistical analysis (0.5 s to 4.0 s). (b) Mean ERD% during MI with the paretic and nonparetic sides. Topographies show the grand average ERD% for the time interval of interest. Topographic data for left paretic patients (controls) were horizontally flipped at the midline, such that the ipsilesional hemisphere is always shown on the left. Please note that in the controls, the term “paretic side” refers to the same side in the healthy control participant as the actual paresis side in the matched stroke patient. Red points indicate the two electrode positions (C3 and C4).

Results of the ERD analysis are depicted in Figure 3. In the upper panel, ERD time-frequency plots across subjects are shown for MI with the paretic hand. As can be seen, a clear reduction of power from 8 to 30 Hz relative to MI onset was observed in both groups. Similar ERD patterns were also observed in the other experimental conditions and are summarized in the two lower panels. As can be seen, throughout conditions, ERDs were evident not only for the hemisphere contralateral to the hand for which MI was performed but also for the ipsilateral side. However, whereas positive ERD differences between the contra- and ipsilateral hemispheres were found in the two control group conditions, as well as in the nonparetic stroke condition, a negative ERD difference was found in the paretic stroke condition. A positive ERD difference indicates the expected ERD lateralization towards the hemisphere contralateral to the imagined hand movement, while a negative ERD difference indicates an ERD lateralization towards the hemisphere ipsilateral to the imagined hand movement. It should, however, be noted that the positive ERD difference in the nonparetic control condition was very small. Statistically, ERD lateralization was investigated using a 2 × 2 mixed-model ANOVA. The analysis revealed no significant main effect of Group (*F*(1,37) = 0.00; *p* < .946) or Side (*F*(1,37) = 1.97; *p* = .168), but a significant Group × Side interaction emerged (*F*(1,37) = 5.62; *p* = .023). Pairwise comparisons were significant between the stroke-paretic and stroke-nonparetic conditions (*T*(36) = −3.48; *p* = .001) and between the stroke-nonparetic and control-nonparetic conditions (*T*(37) = 2.36; *p* = .023). A trend was found between the stroke-paretic and control-paretic conditions *T*(37) = −1.79; *p* = .080), and no effect was found between the control-paretic and control-nonparetic group conditions *T*(36) = 0.78; *p* = .439).

### 3.2. Dependency of MI on Sensitivity and Motor Deficit

**Figure 4:**
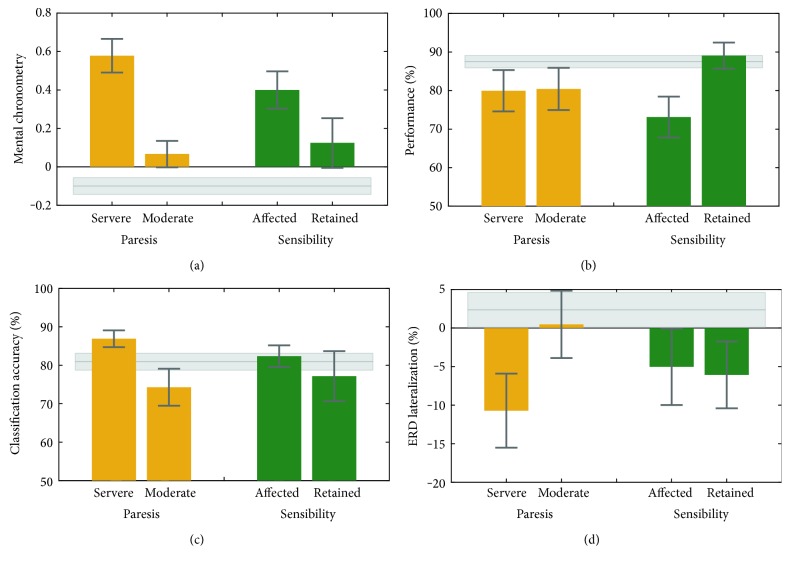
MI performance for patients with and without sensitivity impairment and with moderate or severe paresis. Error bars represent one standard error. Grey shaded area represents control group performances.

Our second question was whether MI impairments are related to sensitivity loss and degree of paresis. Analyses were restricted to the paretic side. Results are presented in Figure 4. The NHPT (Figure 4(a)) indicated that severely paretic patients showed a higher MC score (*M* = 0.57; SD = 0.21) than moderately paretic patients (*M* = 0.07; SD = 0.20) (*t*-test *t*(13) = 4.66; *p* < .001). That is, severely paretic patients performed the task during MI considerably faster than during physical execution. Moderately affected patients tended to do this as well, but to a smaller degree. No significant difference was found regarding the sensitivity comparison (*t*(13) = 1.73; *p* = .106).

LLT performances are illustrated in Figure 4(b). Whereas the degree of paresis did not affect LLT performance (*t*(16) = −0.06; *p* = .952), a significant effect was found in the sensibility comparison (*t*(16) = −2.38; *p* = .029). Here, patients with sensitivity loss had a significantly lower LLT performance (*M* = 73.12; SD = 15.18%) than patients without sensitivity loss (*M* = 89.06%; SD = 17.29%).

The MI-based neurofeedback classification accuracies (Figure 4(c)) were significantly higher (*t*(15) = 2.28; *p* = .037) in severely paretic (*M* = 86.90%; SD = 6.18%) than moderately paretic patients (*M* = 74.28%; SD = 14.42) but did not significantly differ in the sensitivity comparison (*t*(15) = 0.81; *p* = .425).

For ERD lateralization (Figure 4(d)), no significant effect was found for the degree of paresis (*t*(17) = −1.71; *p* = .105) or for the sensory deficit (*t*(17) = 0.14; *p* = .887).

### 3.3. Relationship between the Measures

Results of the correlation analyses are summarized in Table 3. In the nonparetic stroke condition, a significant positive correlation (*r* = .643; *p* = .007) was found between the MC score and LLT performance. That is, in stroke patients but only for the nonparetic side, high LLT performance was associated with imagining a movement faster than executing it. Moreover, for the same condition, a negative correlation (*r* = −.581; *p* = .018) between the MC score and ERD lateralization was found. This correlation reflects that a low ERD lateralization during MI with the nonparetic side was associated with imagining a movement faster than executing it. No significant correlations were found for any of the remaining comparisons.

## 4. Discussion

Aiming to assess the viability of MI as diagnostic or rehabilitative approach following stroke, we investigated to which extended different aspects of MI are preserved following postacute stroke. Behavioral and neural correlates of MI were assessed in three objective MI tasks with a group of stroke patients and age-matched healthy controls.

Our first research question asked whether any of the MI tasks used in the present study indicated an impairment in stroke patients and, if so, whether the impairment was specific to the paretic side. For the NHPT, we found that whereas in the healthy subjects the MC score was slightly below zero on either side, in the stroke patients, it was below zero on the nonparetic side but clearly above zero on the paretic side. That is, on the nonparetic side, MI was slightly slower than motor execution, whereas on the patient's paretic side, MI was clearly faster than motor execution. This finding is in line with the results of a previous study [25], where a similar mental chronometry task was conducted with stroke patients. Dettmers et al. [25] found that on the patient's nonparetic side, MI and motor execution took about the same duration, whereas on the paretic side, the duration for MI was typically much shorter than actual motor execution. Different aspects may account for faster MI than motor execution on the patient's paretic side. First, it may be that patients underestimate the time that is needed for physically conducting the motor task with a paretic limb. That is, although they are already in their postacute or chronic stroke phase, their mental movement trajectory still represents their prestroke movement capacity. In this case, the high MC scores would be predominantly caused by the slowing of motor execution. Second, it could also be that as a result of the stroke, or nonuse of the limb, conducting MI with a paretic limb is less detailed in phenomenal experience and therefore performed faster.

Regarding the LLT, we found that stroke patients generally performed this mental rotation task less accurately than controls. To our knowledge, this is the first study showing such general LLT accuracy drop in postacute stroke patients. Other studies, however, have reported similar LLT accuracy drops for earlier stroke stages or under more specific circumstances. De Vries et al. [40] for instance found a LLT accuracy drop in three, but not six, weeks after stroke, whereas Daprati et al. [41] reported a LLT accuracy drop in right-, but not left-, lesioned stroke patients. On the other hand, there are some studies that found no LLT accuracy differences between stroke patients and controls at all [11], including one of our own studies [12]. How can these conflicting results be explained? One potential reason might be the different stimulus materials used. For instance, whereas in our former study the presented limb pictures were only rotated along one rotational axis, in the present study, the stimulus material was more complex and therefore perhaps more sensitive for detecting impairments in mental rotation ability. Another difference was the response mode being used. Whereas in the present study participants responded verbally, in the study by Liepert et al. [12], responses were collected through button presses with the nonparetic hand. This suggests that the verbal response mode might be more sensitive for detecting mental rotational impairments than the manual response mode (for a comparison of both response modes, see [28]).

Interestingly, none of the stroke studies including the present one reported a paresis side-specific LLT performance reduction. This suggests that after stroke, mental limb rotation is impaired, but very comparable for paretic and nonparetic sides. One reason for this could be that at least some of the representational networks required for mental limb rotation are effector independent (i.e., are recruited by left- and right-limb mental rotations) and that if one of these structures is impaired, deficits can occur irrespective of the side of paresis. This interpretation would be in line with “motor equivalence” studies demonstrating that movements learnt by one effector can also often be performed in a remarkably similar manner by another effector [42–44].

Regarding neurofeedback performance, most of our participants achieved a classification accuracy above the statistical chance level. That is, in most participants, an ERD pattern emerging from MI was evident not only across trials as reflected in the ERD results but also at the single trial level. This provides clear electrophysiological single-trial evidence that in general, patients and healthy controls followed the instructions and were able to perform the MI task. Notably, a difference in the MI EEG-based neurofeedback performance was neither found between the patient and control groups nor between the paretic and nonparetic sides. Those stroke patients which had apparently no bigger difficulty in following the task instructions with their paretic than with their nonparetic side might indicate that either they never lost the ability to imagine the requested movement with their paretic hand or they regained this ability through poststroke cortical reorganization [45]. Although our cross-sectional design does not allow to directly address this question, our ERD results provide some indication for the latter possibility.

The control group showed a bilateral to contralateral ERD pattern during MI. In contrast, the stroke group was characterized by a predominantly contralateral ERD pattern for MI with the nonparetic side and a predominantly ipsilateral ERD pattern for MI with the paretic side. Similar results, especially with respect to a poststroke predominance of the contralesional hemisphere, have been reported in previous MI and motor execution studies [46–48]. Such predominance has been attributed to a compensatory cortical reorganization after stroke, in which the contralesional sensorimotor areas take over the motor functions of their ipsilesional counterparts [45, 49, 50]. That the altered lateralization pattern observed in the present study did not negatively affect classification accuracies is likely due to the fact that even though CSP filters were chosen based on physiological plausibility, the classification algorithm as used here is blind regarding the laterality of the classified brain activity. That is, accuracies will be good as long as there is a reliable difference between baseline and MI task segments even if this difference is most reliable at ipsilateral, contralesional scalp sites.

While early work on neuroplasticity focused on the advantageous aspects of cortical reorganization for motor rehabilitation, a more recent work has also revealed maladaptive patterns [50, 51]. It is widely accepted nowadays that a compensatory, stroke-induced overusage of the contralesional hemisphere may further strengthen the maladaptive underusage of the ipsilesional hemisphere [22]. Evidence in favor of this account comes for instance from several longitudinal neuroimaging studies, showing that an initial predominantly ipsilesional pattern of brain activity in motor tasks is associated with a better motor recovery than a predominantly contralesional pattern (for reviews, see [52, 53]).

Our second research question asked whether MI impairment was related to sensitivity loss or severity of the paresis. An influence of paresis severity was found for the NHPT and the MI-based neurofeedback task. For the NHPT, it was found that moderately paretic patients exhibited a lower MC score—that is, a stronger temporal isochronism between MI and motor execution—than the severely paretic patients. This result extends our above NHPT finding in showing that the extent of MC impairment depends on the degree of paresis. No significant difference in MC was found between our patients with sensory loss and those without. This is in contrast to some of our own former studies where we found worse mental chronometry performances in those patients with sensory loss [12, 15, 25] but is most likely the result of low statistical power for the present analysis due to small sample sizes in the two subgroups.

Concerning the MI EEG-based neurofeedback performance, it was found that more severely paretic patients performed better than moderately paretic patients. This finding is incompatible with the embodied cognition account according to which offline cognition including MI is body based and requires physical action [54]. We are not aware of any other study investigating the influence of paresis severity on MI-based neurofeedback performance. For this reason and because in the present study subgroups were small, this observation should be interpreted with due care. One could speculate that the two subgroups of patients differ not only in their paresis severity but also in their lesion pattern. We explored the idea that the severely paretic patients had more circumscribed lesions directly affecting the descending motor pathways (e.g., lesions in the capsula interna), whereas in the moderately paretic patients, the descending motor pathways remained intact, but the sensorimotor areas themselves became lesioned. As a result, the more severely paretic patients with the intact sensorimotor areas would have been able to produce a more pronounced and consistent ERD pattern than the moderately paretic stroke patients without intact sensorimotor areas. However, our admittedly coarse-grained infarct categorization into cortical, subcortical, and mixed (see Table 2) does not support this view. Rather, it indicates that the moderately paretic stroke patients tended to have subcortical strokes, whereas the severely paretic patients tended to have mixed strokes. Future multimodal studies combining MI EEG neurofeedback with structural and functional MRI will be important to investigate the relationship between MI EEG neurofeedback performance and type, location, and size of the lesion.

An influence of the sensory deficit was only found for the LLT. Patients with sensory loss performed worse than those without. This finding goes in line with previous experimental work showing that short-term upper-limb deafferentation by regional anesthesia leads to a drop in LLT performance [55]. It should, however, be noted that in this former study, the sensory loss was experimentally induced shortly before LLT conductance and in our study, the stroke-induced sensory loss already existed for several weeks to months. The present finding therefore extends the previous one by showing that a LLT performance drop is still observable after long-term sensory loss.

Finally, we conducted a correlation analysis to evaluate how performance in the different MI tasks is related. Only few significant correlations between the different MI measures were found, resulting in the overall impression that the different measures were not strongly related. Although a lack of convergence between different MI measures has been reported before [11, 56–58], we had expected at least some associations in the healthy control group. As it is, the absence of correlations supports the view that MI is not a unitary cognitive function but comes along in many different facets [11, 59].

## 5. Conclusion

In the present study, we investigated different aspects of MI in postacute stroke patients. Even though differences between the stroke and control groups were observed for most MI measures, only some of these differences were specific to the paretic side. MC scores and neurofeedback performances were found to differ for patients with mild and severe paresis. The absence or presence of sensitivity loss was related to mental rotation task performance. No clear pattern emerged from correlating the obtained MI measures. Apparently, MI is not a unitary cognitive function and one MI test alone may not be sufficient to fully assess a stroke patient's or even a healthy person's MI abilities. This conclusion underlines the need for MI assessment tools that take into consideration this diversity.

From a therapeutic perspective, it is remarkable that about 80% of the stroke patients were able to perform the EEG neurofeedback task without much training. This supports the hope that MI as a “backdoor to the motor system” may mature into a therapeutic option in particular for patients with severe paresis. However, our ERD data suggest that the good neurofeedback performance for the paretic side resulted from a potentially maladaptive activation pattern. Clearly, this possibility and its consequences should be taken into consideration in the design of future MI neurofeedback training studies.

## Conflicts of Interest

The authors declare no competing financial interests.

## Figures and Tables

**Table 1:** Demographic data of stroke patients and controls.

	Stroke (*N* = 20)	Control (*N* = 20)
Sex (male : female)	11 : 9	11 : 9
Age (SD)	59.1 (9.94)	60.1 (7.67)
Handedness (left : right)	3 : 17	1 : 19
Motor dexterity	2.00 (1.11)	1.00 (0.10)
Sensitivity (affected : not affected)	12 : 8	—
Months since stroke (SD)	9.85 (12.03)	—
Infarct side (left : right)	8 : 12	—
Infarct location (cortical : subcortical : mixed)	2 : 9 : 9	—
Paresis side (left : right)	12 : 8	—
MOCA	22.5 (5.63)	—

**Table 2:** Demographic and clinical characteristics of the different subgroups.

	Sensitivity		Paresis	
	Affected (*N* = 12)	Retained (*N* = 8)	Severe (*N* = 10)	Moderate (*N* = 10)
Sex (male : female)	4 : 8	7 : 1	5 : 5	6 : 4
Age (SD)	58.66 (11.79)	59.75 (7.00)	59.50 (12.25)	58.70 (7.63)
Months since stroke (SD)	9.43 (9.65)	13.43 (15.90)	10.92 (12.46)	11.14 (12.79)
Infarct side (left : right)	5 : 7	3 : 5	4 : 6	4 : 6
Infarct location (cortical : subcortical : mixed)	1 : 5 : 6	1 : 4 : 3	1 : 3 : 6	1 : 6 : 3

**Table 3:** Results of correlation analysis using Pearson's *r* correlations.

	Stroke (*N* = 20)		Control (*N* = 20)	
Pair of correlation	Paretic	Nonparetic	Paretic	Nonparetic
Mental chronometry versus LLT performance	.145	.643^∗∗^	−.413	.038
Mental chronometry versus classification accuracy	.028	−.265	−.099	−.293
Mental chronometry versus ERD lateralization	−.203	−.581^∗^	.120	−.280
LLT performance versus classification accuracy	.166	−.090	−.161	.046
LLT performance versus ERD lateralization	.355	−.265	−.354	−.234
Classification accuracy versus ERD lateralization	−.355	.408	.110	−.024

^∗^ *p* < 0.05; ^∗∗^*p* < 0.01.
